# Resting salivary flow independently associated with oral malodor

**DOI:** 10.1186/s12903-016-0255-3

**Published:** 2016-07-19

**Authors:** N. Suzuki, A. Fujimoto, M. Yoneda, T. Watanabe, T. Hirofuji, T. Hanioka

**Affiliations:** Department of Preventive and Public Health Dentistry, Fukuoka Dental College, 2-15-1 Tamura, Sawara-ku Fukuoka, 814-0193 Japan; Department of General Dentistry, Fukuoka Dental College, Fukuoka, Japan

**Keywords:** Oral malodor, Mucosal moisture, Periodontitis, Resting saliva, Stimulated saliva, Tongue coating

## Abstract

**Background:**

Dryness of the oral cavity is considered one cause of oral malodor. However, it is unclear which of the factors regulating the wetness of the oral cavity are involved in oral malodor development. This study investigated the effects of salivary flow and oral mucosal moisture on oral malodor.

**Methods:**

The study population comprised 119 patients (48 men and 71 women, mean age of 50.6 ± 15.4 years) with complaint of oral malodor. After the oral malodor level had been evaluated by the organoleptic test and gas chromatography, the rates of stimulated saliva and resting saliva and the moisture levels of the tongue and buccal mucosa were measured. The plaque index, bleeding on pocket probing, probing pocket depth, and tongue coating score were also assessed. Strong oral malodor was defined as an organoleptic test score of ≥3.

**Results:**

The flow rate of resting saliva in women was significantly lower than in men. The flow rate of resting saliva and the moisture levels of the tongue and buccal mucosa showed significant negative correlations with age. The flow rate of resting saliva was significantly lower in patients with strong oral malodor than in those with no or weak oral malodor. The flow rate of stimulated saliva and the moisture levels of the tongue and buccal mucosa had no relationship with strong oral malodor. Logistic regression analysis showed that a ≥5-mm probing pocket depth with bleeding on pocket probing, an increased tongue coating score, and decreased resting salivary flow were strong explanatory factors in clinical findings for oral malodor.

**Conclusion:**

This study suggests that the flow rate of resting saliva is a significant modulating factor for oral malodor.

**Electronic supplementary material:**

The online version of this article (doi:10.1186/s12903-016-0255-3) contains supplementary material, which is available to authorized users.

## Background

Oral malodor is a common problem in humans. It typically originates directly from the oral cavity secondary to periodontitis, tongue debris, poor oral hygiene, deep caries, inadequately fitted restorations, or endodontic lesions [1–3]. The major compounds that contribute to oral malodor are volatile sulfur compounds (VSCs) such as hydrogen sulfide and methyl mercaptan, which are primarily the result of the microbial metabolism of amino acids in local debris in the oral cavity [4, 5]. It has been suggested that dryness of the oral cavity induces the expression of volatile [6] or non-sulfur-containing gases [7]. Many antibacterial factors are present in mucosal secretions of the oral cavity; in addition, saliva has a self-purifying effect. The flow of mucosal secretions in the oral cavity can be considered to be a factor that influences oral malodor.

Assessments of stimulated salivary flow and resting salivary flow are used to evaluate the flow of mucosal secretions in the oral cavity [8, 9]. They differ in terms of viscosity and composition [10]. Twenty percent of resting salivary flow is derived from the parotid gland, 65 % from the submandibular, 7–8 % from the sublingual, and <10 % from numerous minor glands [10]. Upon stimulated salivary flow, there are marked changes in the proportional contributions from each gland, with the parotid contributing more than 50 % of total salivary secretions. In previous studies, different conclusions were drawn about the relationship between these types of salivary flow and oral malodor [11–13].

The mucosal moisture level has been used to evaluate the dryness of the oral cavity. It was reported that the mucosal moisture level of the tongue and the resting salivary flow significantly decreased after sialoadenectomy in guinea pigs [14]. The moisture levels of the tongue and buccal mucosa of patients with subjective symptoms of dry mouth were also found to be significantly lower than those of patients without such symptoms [15]. However, few studies have evaluated the relationship between mucosal moisture levels and oral malodor. In addition, the relationship between the salivary flow and mucosal moisture levels has seldom been studied.

In the current study, the stimulated salivary flow, resting salivary flow, and mucosal moisture levels in the oral cavity were measured in patients with the complaint of halitosis to evaluate the relationship among these factors and the relationships between these factors and oral malodor under the null hypothesis that mucosal secretions in the oral cavity have no relation to oral malodor.

## Methods

### Study population

The study population comprised 119 patients (48 men and 71 women, mean age of 50.6 ± 15.4 years) with complaint of halitosis who presented at the Oral Malodor Clinic of Fukuoka Dental College Medical and Dental Hospital between September 2012 and December 2015. They had not taken antibiotics within 3 months and had no otorhinolaryngological illness or metabolic disease.

### Malodor assessment

The severity of oral malodor in each patient was determined using an organoleptic test (OLT) and gas chromatography. Malodor assessment and clinical examination including tests of salivary flow and mucosal moisture level were performed at least 5 h after eating, drinking, chewing, smoking, and brushing or rinsing of the mouth. The OLT scores were estimated by two of three evaluators using a scale of 0 to 5 [16], and the mean of the scores given by the evaluators was used. The presence of OLT scores ≥2 among the three evaluators always exceeded 75 % (k = .50). Gas chromatography (model GC2014; Shimazu Works, Kyoto, Japan) was used to measure the concentrations of hydrogen sulfide (H_2_S), methyl mercaptan (CH_3_SH), and dimethyl sulfide (CH_3_SCH_3_) in mouth air. The value for total VSCs was defined as the sum of the H_2_S, CH_3_SH, and CH_3_SCH_3_ concentrations. The threshold level for genuine halitosis was defined as an OLT score of ≥3 after rounding off to the nearest integer [16].

### Measurements of salivary flow and mucosal moisture levels

The flow rate of stimulated saliva was measured using the chewing gum test [17]. The participants were asked to spit into a vessel throughout a 5-min collection period while chewing gum to collect saliva (Chekbuf; Morita, Osaka, Japan). The flow rate of resting saliva was measured in accordance with a previous study [18]. Briefly, one cotton roll was placed between the tongue and the lower anterior teeth in front of the sublingual and submaxillary duct apertures to absorb saliva at the lower part of the mouth. After 1 min, each cotton was removed and weighed individually to determine the increase in weight, corresponding to the saliva collected at the lower part of the mouth. An electronic device (Mucus®; Life Co., Saitama, Japan) that measures the moisture of the submucosal layer of the tongue (about 50 μm under the mucosal surface) was used to measure the moisture levels of the tongue and buccal mucosa [14]. This measurement was performed after placing the sensor cover over the device sensor in accordance with the manufacturer’s instructions. The sites of measurement were the dorsum of the tongue 10 mm from the apex linguae and the right and left buccal mucosa 10 mm from the corner of the mouth. The moisture level of the buccal mucosa was calculated as the mean of the right and left values.

### Clinical examination

Periodontal health, plaque control, and the degree of tongue coating were evaluated as clinical parameters related to oral malodor in addition to the wetting force in the oral cavity. Periodontal health was assessed using the average probing pocket depth (PPD) and the percentage of bleeding on probing (BOP) sites. PPD and BOP were measured at six points around each tooth in all participants. The presence of ≥5-mm PPD with BOP was recorded as the presence of periodontitis [19]. Plaque control was evaluated using the Silness and Löe Plaque Index (PlI) [20]. The degree of tongue coating was determined based on the tongue coating score (TCS) using a scale of 0 to 4 [21].

### Statistical analysis

The Mann–Whitney *U* test and the χ^2^ test were used to compare clinical parameters between men and women and between OLT scores of <3 and ≥3. Pearson correlation coefficients were calculated to evaluate the relationships between age and parameters related to salivary flow and mucosal moisture. Partial correlation coefficients were calculated to evaluate the relationships between age and the parameters that determine the wetness and moisture of the oral cavity. A multivariable logistic regression model was conducted to determine clinical parameters strongly related to oral malodor: such parameters included PlI, the presence of a ≥5-mm PPD with BOP, TCS, and the flow rate of resting saliva. All statistical analyses were performed using SPSS software (version 22.0; SPSS Japan, Tokyo, Japan). A *P* value of <0.05 was considered to reflect statistical significance.

## Results

### Profiles of the study population

The profiles of the study population are shown in Table 1. The raw data are provided in the Additional file 1. There was no significant difference in the parameters between men and women except for the flow rate of resting saliva. Specifically, women had a significantly lower flow rate of resting saliva than men (*P* = 0.049). The flow rate of stimulated saliva in women was also lower than in men, but there was no significant difference (*P* = 0.134). The moisture levels of the tongue and buccal mucosa did not differ between men and women.

**Table 1:** Profiles of the subjects. Data are reported as median and lower and upper quartile values

Parameters	Total (*n* = 119)	Men (*n* = 48)	Women (*n* = 71)
Age (y)	52 (39–63)	49 (40.8–63)	55 (37.5–63.5)
OLT score	2.5 (2.0–3.0)	2.5 (2.0–3.0)	2.5 (2.0–3.0)
VSCs (ng/10 mL)	4.8 (2.0–7.9)	4.4 (1.9–7.1)	5.1 (2.0–9.3)
No. of teeth	28 (25–28)	28 (26–28)	28 (24–28)
PlI	0.5 (0.3–0.6)	0.5 (0.3–0.6)	0.4 (0.3–0.6)
TCS	2.0 (1.0–2.0)	2.0 (1.0–2.0)	2.0 (1.0–2.0)
Ave. PPD (mm)	3.0 (3.0–3.2)	3.0 (3.0–3.3)	3.0 (3.0–3.2)
BOP (%)	6.5 (2.4–11.1)	6.5 (2.4–9.8)	6.5 (2.4–11.6)
Stimulated salivary flow (mL/5 min)	7.0 (5.0–11.0)	7.5 (5.0–12.5)	6.0 (4.8–11.0)
Resting salivary flow (mL/min)	0.13 (0.06–0.20)	0.15 (0.10–0.21)*	0.12 (0.04–0.20)*
Moisture of tongue	29.9 (27.4–31.2)	29.6 (27.2–31.4)	29.9 (28.0–31.1)
Moisture of buccal mucosa	30.0 (28.1–31.5)	30.3 (28.2–31.6)	30.0 (28.1–31.3)

Mann–Whitney *U* test. * *P* <0.05. *OLT* organoleptic test, *VSCs* volatile sulfur compounds, *PlI* plaque index, *TCS* tongue coating score, *PPD* probing pocket depth, *BOP* bleeding on probing

### Correlations between age and parameters related to wetness and moisture of the oral cavity

The correlations between age and the parameters related to wetness and moisture of the oral cavity were analyzed. The flow rate of resting saliva showed a negative correlation with age (*r* = −0.257, *P* = 0.005), whereas the stimulated salivary flow did not (*r* = −0.088, *P* = 0.339). The moisture levels of the tongue and buccal mucosa each showed a negative correlation with age (tongue and age, *r* = −0.222, *P* = 0.015; buccal mucosa and age, *r* = −0.245, *P* = 0.007).

### Correlations among parameters related to wetness of the oral cavity after adjusting for age

The correlation coefficients between the parameters related to the wetness and moisture of the oral cavity after adjusting for age were analyzed. There was a weak positive correlation between the stimulated salivary flow and resting salivary flow (*r* = 0.230, *P* = 0.012). There was a moderate positive correlation between the moisture levels of the tongue and buccal mucosa (*r* = 0.531, *P* <0.001). There was no relationship between salivary flow and mucosal moisture.

### Comparison of the parameters based on oral malodor

Table 2 shows a comparison of the parameters between no or weak oral malodor (OLT score of <3) and strong oral malodor (OLT score of ≥3). The concentration of VSCs, which are major compounds that substantially contribute to oral malodor, was significantly higher in the subjects with strong oral malodor than in those with no or weak oral malodor (*P* <0.001). Regarding clinical parameters, PlI (*P* = 0.035), TCS (*P* <0.001), PPD (*P* = 0.024), and BOP (*P* = 0.005) were significantly higher in the subjects with strong oral malodor than in those with no or weak oral malodor. The presence of ≥5-mm PPD with BOP, which was defined as the presence of periodontitis, was also significantly higher in the subjects with strong oral malodor (*P* <0.001). In contrast, the flow rate of resting saliva was significantly lower in the subjects with strong oral malodor than in those with no or weak oral malodor (*P* = 0.005). The flow rate of stimulated saliva and the moisture levels of the tongue and buccal mucosa did not differ between the two groups.

**Table 2:** Correlation of parameters based on oral malodor. Data are reported as median and lower and upper quartile values

Parameters	OLT score < 3 (*n* = 58)	OLT score ≥ 3 (*n* = 61)
Age (y)	48.5 (40.3–61.5)	56.0 (39.0–65.0)
VSCs (ng/10 mL)	2.5 (1.1–4.5)**	7.3 (4.8–13.2)**
No. of teeth	28 (26–28)	28 (23–28)
PlI	0.4 (0.3–0.6)*	0.5 (0.4–0.6)*
TCS	1.0 (1.0–2.0)**	2.0 (1.0–2.0)**
Ave. PPD (mm)	3.0 (2.9–3.1)*	3.0 (3.0–3.4)*
BOP (%)	4.6 (0.8–8.5)**	7.7 (3.1–13.2)**
Presence of ≥5-mm PPD with BOP	11 (19.0 %)**	30 (49.2 %)**
Stimulated salivary flow (mL/5 min)	8.3 (5.0–11.4)	6.0 (5.0–10.0)
Resting salivary flow (mL/min)	0.15 (0.11–0.26)**	0.09 (0.04–0.19)**
Moisture of tongue	29.1 (26.9–31.2)	30.2 (28.5–31.1)
Moisture of buccal mucosa	29.4 (27.9–31.6)	30.3 (28.4–31.3)

Presence of ≥5-mm PPD with BOP was analyzed by *χ* ^2^ test

The other parameters were analyzed by the Mann–Whitney *U* test

* *P* <0.05, ** *P* <0.01

(The *P* value of the number of teeth was 0.05)

Table 3 shows a logistic regression model of the characteristics in clinical findings associated with strong oral malodor (OLT score of ≥3). The subjects with a ≥5-mm PPD with BOP were 3.17 times (317 %) more likely to present with strong oral malodor than those without a ≥5-mm PPD with BOP (*P* = 0.014). The odds ratio calculated for the subjects with strong oral malodor was 2.41 (241 %) when compared with the other subjects, in terms of an increase in TCS of 1 point (*P* = 0.003). The odds ratio calculated for the subjects with strong oral malodor was significantly lower (odds ratio = 0.04, 4 %) than that for the other subjects, in terms of a 0.1-mL/min increase in the resting salivary flow (*P* = 0.039). PlI were not associated with strong oral malodor.

**Table 3:** Logistic regression model of characteristics associated with strong oral malodor in clinical findings (OLT score ≥ 3)

Parameters	Odds ratio	95 % CI	*P* value
Increase of 0.1 in P1I	2.13	0.33–13.7	0.428
Presence of ≥5-mm PPD with BOP	3.17	1.26–7.99	0.014
Increase of TCS by 1 point	2.41	1.36–4.26	0.003
Increase of resting salivary flow by 0.1 mL/min	0.04	0.00–0.85	0.039

*PlI* plaque index, *PPD* probing pocket depth, *BOP* bleeding on probing, *TCS* tongue coating score

## Discussion

The primary aim of the study was to investigate the effects of salivary flow and mucosal moisture on oral malodor. The results revealed that resting salivary flow is an important modulating factor of oral malodor.

Salivary flow is considered to be affected by age and sex. The functional failures of the salivary glands cause a reduction in salivary flow and increase in dryness, which are common in elderly people [22]. A recent meta-analysis of salivary flow rates in young and older adults reported that whole and submandibular and sublingual salivary flow rates were reduced significantly in older participants, whereas parotid and minor gland salivary flow rates were not significantly reduced with increasing age [8]. The results of the current study are consistent with these findings: the flow rate of resting saliva (i.e., submandibular and sublingual saliva) showed a negative correlation with age, whereas the flow rate of stimulated saliva had no correlation with age. In addition, a sex difference was observed in the flow rate of resting saliva. Inoue et al. [23] investigated the resting salivary flow rate and salivary gland size in healthy young adults and reported that both were smaller in women than in men.

The moisture level of the skin is commonly used to measure aging. The moisture level of the oral mucosa reportedly decreased with aging in a study that assessed the midline of the lower labial mucosa [24]. However, the changes in the moisture levels of the tongue and buccal mucosa with age or sex were unclear. In the current study, the moisture levels of the tongue and buccal mucosa were negatively correlated with age. Sex had no effect on the moisture levels of the tongue and buccal mucosa.

The major causes of oral malodor are tongue coating, poor oral hygiene, and periodontitis; therefore, the clinical parameters related to these, such as TCS, PlI, average PPD, and BOP, are associated with strong oral malodor. Concerning the wetness and moisture of the oral cavity, the flow rate of resting saliva in subjects with oral malodor was significantly lower than in subjects with no or weak oral malodor. Stimulated salivary flow and the moisture of the tongue surface and buccal mucosa were not associated with strong oral malodor. Several studies investigated the relationship between salivary flow and oral malodor. Koshimune et al. [11] obtained results similar to those in the current work. On the other hand, another report stated that the flow rate of resting saliva did not differ between subjects with oral malodor and those with no or weak oral malodor [12]. The resting whole saliva was collected by a draining method in those studies, whereas the saliva collected in the current study was limited to the submandibular and sublingual saliva obtained by the cotton roll method. Several other studies also reported that the stimulated salivary flow is not associated with oral malodor [13].

Few studies have assessed the relationship between oral mucosal moisture and oral malodor. The moisture checker used in the current study measures the moisture of the submucosal layer, about 50 μm under the mucosal surface, and is useful for evaluating the dryness in patients with xerostomia [14, 15]. The patients in the current study did not have xerostomia, and their moisture levels of the tongue and buccal mucosa were higher than the threshold of dryness (=25) defined by the manufacturer. The moisture levels of the tongue and buccal mucosa may not be associated with strong oral malodor in generally healthy populations. This instrument may be useful for investigating the oral malodor in patients with xerostomia.

Logistic regression analysis included clinical parameters that were significantly related to strong oral malodor (TCS, PlI, the presence of a ≥5-mm PPD with BOP, and the flow rate of resting saliva). It revealed that TCS, the presence of a ≥5-mm PPD with BOP, and the flow rate of resting saliva were important modulating factors for strong oral malodor. The method of saliva testing used in the current study is simple and can be performed in a short time. It may thus be suitable for use in clinical practice. In addition, our results indicate that it is advisable to consider resting salivary flow as an important modulating factor in future research concerning oral malodor.

The current study is limited by the numbers of men and women and the age groups of the subjects. The numbers of men and women were 48 and 71, respectively. Women tend to present with the complaint of halitosis more often than men [21, 25]. More than half of the subjects (53.8 %) were ≥50 years of age in the current study, and there were very few young people: the proportion under 30 years of age was only 10.9 %. Therefore, it can be said that analyses were performed on the middle-aged and elderly age groups. These shortcomings should be considered when interpreting the obtained results.

## Conclusions

In conclusion, the results of this study indicate that tongue coating, periodontitis, and the reduction of resting saliva are significant modulating factors in clinical findings for strong oral malodor among generally healthy adults complaining of halitosis in Japan. Appropriate oral hygiene instructions and treatment for these factors may be useful in the management of oral malodor by dentists and dental hygienists.

## Additional file

Additional file 1: The raw data. (PDF 42 kb)
