# Supplementary material for: Resting salivary flow independently associated with oral malodor
Source: BMC Oral Health. 2016 Jul 19;17:23. doi: 10.1186/s12903-016-0255-3 (PMC4950720; doi:10.1186/s12903-016-0255-3)
Supplement: Additional file 1: — The raw data. (PDF 42 kb) [file 12903_2016_255_MOESM1_ESM.pdf]

No	1male 2female	halimeter (ppb)	H2S(ng/10ml)	CH3SH(ng/10ml)	CH3SCH3(ng/10ml)	total VSC(mg/10ml)	OLS0-5	teeth	caries	filling	TCS1-4	Color of tongue 1white 2white-yellow 3 green 99no	PPD >=5mm aith BOP+ 1yes 2no	PPD mm	mobility 1yes 2no	plaque index	BOP%	stimulated saliva ml/5min	resting saliva ml/min	tongue moisture	buccal moisture	smoking 1current 2before 3non
1	1	280	2.07	0.85	0.27	3.19	2.75	28	0	2	2	2	2	3	2	0.33	0.6	15	0.04	32.2	33.7	1
2	1	112	1.544	0.604	0.452	2.6	2.5	28	0	2	3	2	2	3	2	0.29	1.19	17.5	0.21	29.3	30	1
3	2	30	0.054	0.064	0	0.118	0.5	27	0	16	2	2	2	3	2	0.58	1.23	3.5	0.2	26.7	33.3	3
5	2	773	11.104	3.748	0.926	15.778	3.25	23	0	16	2	2	1	3.2	2	0.38	4.35	5	0.13	31	30.2	2
6	2	265	3.438	2.41	1.706	7.554	2	28	0	13	1	2	1	3.1	2	0.54	11.9	14.4	0.15	28.8	26.7	3
7	2	103	1.992	1.188	1.848	5.028	2.5	28	0	19	1	2	2	3	2	0.5	7.74	13	0.26	30.8	31.5	1
8	2	166	1.002	1.14	0.992	3.134	2.25	29	1	18	1	2	1	3.6	2	0.63	27.6	6	0.13	32.5	33.4	3
9	2	437	5.822	2.878	1.834	10.534	3	27	2	13	1	2	1	3.1	1	0.46	2.47	5	0.05	31.3	33.4	3
10	1	35	0	0	0.344	0.344	2	26	0	22	1	1	2	2.9	2	0.67	2.56	6.5	0.13	31	31.6	3
11	2	95	0.104	0.172	0.368	0.644	1.75	23	0	18	1	2	2	3.1	2	0.42	5.07	3	0.12	32.4	31.6	3
12	2	131	1.618	0.444	0.112	2.174	2.5	28	1	20	1	2	2	3	2	0.38	1.19	5.5	0.04	31.1	30.2	3
13	2	49	0.192	0.148	0.322	0.662	1	28	1	5	0	99	2	3	2	0.25	5.56	12	0.31	31.9	29.55	1
14	2	96	2.112	2.07	1.272	5.454	2.25	24	3	11	2	2	1	3.8	2	1.25	54.2	13.75	0.16	28.3	25.4	3
15	1	30	0.088	0.07	0.35	0.508	0.25	21	0	21	1	2	2	3	2	0.17	0	13.75	0.55	26.3	28.2	2
16	1	111	0.47	0.748	0.566	1.784	2	28	0	17	3	1	1	3.46	2	0.33	17.3	15	0.1	25.7	28.2	2
17	2	133	2.332	1.448	0.8	4.58	2	28	0	4	2	2	2	3	2	0.29	0	3.5	0.01	23.75	30	3
18	2	133	4.436	4.55	2.02	11.006	3	23	2	8	2	2	1	4.04	1	1.42	12.3	4.5	0.06	22.45	29.55	3
19	1	162	0.402	3.342	1.036	4.78	3	24	0	17	1	1	1	3.27	1	0.38	18.75	5.5	0.11	21.2	25.75	1
20	1	233	2.106	4.06	0.91	7.076	3	24	0	17	2	1	1	3.79	1	0.5	13.2	4	0.07	23.45	29.35	1
21	2	162	0.464	0.752	0.678	1.894	2.5	31	1	17	1	2	1	3.2	2	0.63	8.6	11	0.02	30.2	27.2	3
22	2	204	4.17	2.476	1.058	7.704	3	25	0	24	1	2	2	3	2	0.42	2.67	7.5	0.11	25.35	28.95	3
23	1	475	3.912	3.114	2.054	9.08	3.5	26	0	2	3	2	1	3.7	2	1	17.3	5	0.2	28.75	30.45	3
24	2	148	3.466	0.294	0.526	4.286	2.75	28	0	11	2	3	2	2.8	2	0.21	3.57	5	0.43	33	32.95	3
25	2	237	7.076	3.242	5.62	15.938	3	28	0	13	2	2	2	3	2	0.5	8.93	6	0.33	28.45	30.35	3
42	1	96	0.528	0.31	0.576	1.414	2.25	29	0	17	1	2	2	3.16	2	0.21	0	2	0.17	26.8	25.6	3
43	2	146	0.984	0.628	0.388	2	1.25	28	0	13	1	1	2	3.05	2	0.54	0.1	5	0.19	29.9	29.1	3
44	2	67	0.0146	0.064	0	0.0786	1.5	32	0	4	1	1	2	3.03	2	0.29	5.21	9	0.26	31.1	30.7	3
45	2	99	0.67	0.276	0.598	1.544	1.75	25	0	8	1	2	1	2.7	1	0.33	1.33	12	0.05	30.9	29.65	3
46	2	183	2.39	3.182	1.692	7.264	2	23	0	17	0	99	2	3.02	2	0.33	0.42	1	0.08	24.8	28.73	3
47	2	145	1.562	0.44	0.286	2.288	2.5	23	0	13	0	99	1	2.78	1	0.5	6.52	2.5	0.16	25.2	31.2	3
48	1	477	3.54	0.938	0.298	4.776	2.5	29	0	16	2	2	1	3.36	2	0.21	12.07	7	0.2	30.4	30.45	1
49	1	272	2.474	0.65	0.374	3.498	1.75	29	0	16	1	2	2	2.93	2	0.5	5.17	7	0.14	30.3	31.5	1
50	1	146	1.09	0.38	0	1.47	1.5	29	0	16	2	2	1	3	2	0.29	7.47	7	0.16	33	32.85	1
51	2	106	9.402	5.466	3.806	18.674	2	28	0	0	1	1	2	3	2	0.5	20.8	12	0.4	32.5	32.65	3
52	2	426	5.852	1.882	0.4	8.134	2.25	30	0	18	2	1	2	3.03	2	0.71	0.56	11	0.13	12.9	31.8	3
53	1	43	0.098	0	0	0.098	1.25	30	0	19	0	99	2	3.05	2	0.42	0	5	0.15	26.45	28.3	2
54	1	217	9.548	2.652	1.92	14.12	2	27	0	12	2	2	2	2.54	2	0.17	2.47	16.25	0.17	30.75	29.4	3
55	2	148	0.928	0.79	0.76	2.478	1.5	28	0	19	1	1	2	2.85	2	0	0	11	0.14	28.4	25.8	3
56	1	45	4.634	2.482	0.936	8.052	3.25	5	0	5	3	2	1	4.7	1	0.5	6.7	13	0.24	29.9	31.4	2
57	1	96	2.892	1.222	0.628	4.742	3	5	0	5	2	2	1	4.4	1	0.5	10	17.5	0.22	30.3	29.4	2
58	2	209	2.192	1.924	2.128	6.244	1.75	28	0	13	2	2	2	3	2	0.29	7.14	11.5	0.64	28.4	28.7	3
59	2	282	2.722	2.018	1.684	6.424	1.75	28	0	13	2	2	2	3	2	0.375	10.1	11	0.2	27	27.9	3
60	2	327	3.32	1.518	1.202	6.04	2	28	0	13	2	2	2	2.75	2	0.25	4.76	11	0.32	31.6	31	3
61	2	107	0.234	0.334	0.598	1.166	2	24	2	4	1	1	2	2.96	2	0.71	20.8	5	0.26	31.5	32.3	3
62	1	165	2.628	0.34	0.208	3.176	1.5	28	2	5	1	2	2	3	2	0.542	0	9	0.13	29.3	29.4	3
63	1	39	0.056	0	0	0.056	2.25	26	0	19	2	2	1	3.33	1	0.46	8.65	5	0.04	25.3	26.7	2
64	1	367	4.512	1.52	0.878	6.91	2.25	31	1	17	1	2	2	2.94	2	0.42	6.45	12.5	0.07	28.2	28.3	3
65	2	155	1.502	0.758	0.492	2.752	1.75	29	0	7	1	1	2	3	2	0.25	1.72	11.1	0.1	26.7	27.2	3
66	2	228	2.218	1.93	0.644	4.792	3.25	17	1	12	2	2	1	2.94	1	0.58	11.8	9	0.01	28.45	26.5	3
67	1	163	1.356	1.294	1	3.65	2.25	27	0	23	1	1	2	3.02	2	0.67	8.02	4.5	0.15	27.5	28	2
68	2	31	0.138	0.044	0	0.182	3	14	0	10	2	2	1	3.21	2	0.375	4.76	4.5	0.03	27.2	26.5	1
69	2	46	0.822	0.338	0.578	1.738	1.25	26	0	21	2	2	2	3	2	0.33	1.28	4	0.1	29.9	30.1	3
70	2	120	0.928	0.634	1.63	3.192	2	28	0	12	0	99	1	3.25	2	0.92	13.1	11.25	0.66	31.4	33.8	3
71	1	283	9.696	3.658	1.244	14.598	2.5	28	0	6	2	1	1	3.09	2	0.625	14.9	2.5	0.08	31	31.2	2
72	2	76	0.644	0.262	0.55	1.456	1.25	28	0	14	0	99	2	2.93	2	0.42	9.52	4.5	0.12	30.2	29.4	3
73	2	356	4.872	1.5	0.768	7.14	2.75	23	0	14	2	2	2	3.43	2	0.21	4.34	6	0.01	29.65	28.35	3
74	1	66	0.684	0.138	0.226	1.048	1	25	1	15	1	1	2	3.06	2	0.17	3.3	12.5	0.12	25.5	29	3
75	2	213	3.564	1.95	0.968	6.482	3	28	0	7	0	99	2	4.75	2	0.79	80.4	5.5	0.07	30.5	29.45	3
76	1	288	4.142	1.064	1.126	6.332	2.5	28	0	15	1	1	2	3.04	2	0.42	2.98	5.5	0.05	30.3	30.05	2
77	2	387	10.786	13.802	7.016	31.604	4	17	0	15	3	2	1	3.38	2	1.125	24.5	5	0.01	30.7	26	3
78	1	212	4.25	0.862	1.24	6.352	3	26	0	21	2	2	1	3.58	1	0.54	7.69	15.6	0.12	27.3	26.25	1
79	1	168.5	4.14	0.602	0.822	5.564	2.5	29	0	8	2	2	1	3.03	2	0.58	9.77	8	0.23	25.7	27.45	3
80	2	321	18.218	2.76	1.18	22.158	3	32	0	12	1	2	2	3.03	2	0.625	10.9	4	0.05	30.9	30.8	2
81	2	204	7.662	1.4	1.386	10.448	2.75	28	0	10	2	2	2	3	2	0.21	2.97	5	0.04	28.9	27.4	3
82	2	107	3.952	0.584	0.604	5.14	2	25	0	17	1	1	1	3.16	1	0.08	2.67	6	0.06	26.85	23.5	3
83	2	71	0.934	0.466	0.922	2.322	2.5	28	0	15	1	1	2	2.96	2	0.58	13.7	7	0.06	30.2	31	3
84	1	300	5.21	0.996	0.7	6.906	2.5	28	0	15	1	1	2	3	2	0.83	2.38	7	0.22	33	32.25	3
85	1	57	0.566	0.42	0.94	1.926	1	28	1	22	1	1	2	3.21	2	0.5	14.9	8	0.4	31.2	31.6	3
86	1	40	0.064	0	0	0.064	2	28	0	13	2	2	2	2.86	2	0	6.55	4	0.3	26.9	26.9	1
87	1	846	14.626	3.514	1.59	19.73	2.5	28	0	6	2	2	2	2.96	2	0.5	8.92	3.5	0.15	32.95	32.3	3
88	2	247	7.244	4.038	1.778	13.06	3	28	0	14	2	2	2	2.77	2	0.375	7.14	10	0.2	32.1	31.3	3
89	1	179	1.906	0.844	0.884	3.634	1.75	26	0	11	1	2	2	2.75	2	0.25	9.6	7	0.2	28.75	32	3
90	2	207	2.864	1.854	0.944	5.662	3	23	0	3	2	1	2	2.91	2	0.21	0	9	0.04	29.6	31.4	3
91	2	75	0.482	0.196	0.258	0.936	2	27	0	8	1	2	2	3	2	0.5	6.79	4.5	0.52	30.8	30.35	1
92	1	433	8.544	2.374	0.894	11.812	2.5	28	0	15	2	1	2	2.96	2	0.83	3.57	8	0.19	31	30.6	3
93	1	77	1.404	0.734	0	2.138	2.75	28	0	2	2	2	2	3	2	0.208	0	21.8	0.11	33.3	30.7	1
94	1	202	1.384	0.738	0.484	2.606	1.75	28	0	6	1	1	2	3.02	2	0.458	6.54	5	0.12	32.2	30.5	3
95	2	67	2.552	0.732	0.584	3.868	2	28	0	11	1	1	2	2.63	2	0.208	0	6	0.22	28	29.4	3
96	2	206	3.636	1.452	0.772	5.86	2	28	0	7	1	1	1	3.93	2	0.625	7.14	8.5	0.36	35.6	33.7	3
97	1	161	2.404	0.946	0.684	4.034	2.25	28	0	15	3	2	2	3	2	0.5	5.36	12	0.08	33.1	32.1	3
98	2	354	7.526	4.992	2.09	14.608	4	18	0	6	3	2	1	3.17	2	0.125	6.48	3.5	0.08	34.1	30.3	2
99	2	90	0.544	0.378	0.316	1.238	3.5	22	0	22	2	2	2	3.02	2	0.708	7.58	9.5	0.12	30.7	30.5	3
100	1	242	2.808	0.778	0.444	4.03	2.5	28	0	15	1	1	2	2.8	2	0.25	1.19	11	0.3	33.25	31.85	3
101	1	116	8.398	8.046	0.748	17.192	3	26	0	22	2	2	1	3.62	2	0.458	7.69	7	0.04	31.8	29.1	3
102	2	174	4.716	1.14	0.656	6.512	2	28	0	12	1	2	2	2.93	2	0.21	0	9	0.18	27.15	28.15	3
103	2	173	2.414	2.744	1.672	6.83	3	28	0	3	1	2	2	3	2	0.5	4.76	15	0.15	29.3	29.1	3
104	2	143	1.17	0.272	0.546	1.988	1.5	28	0	0	0	99	2	2.73	2	0.21	2.38	11	0.3	25.6	25.2	3
105	2	1154	9.124	6.444	2.212	17.78	3.5	28	0	22	3	2	2	3.07	2	0.92	29.2	2	0.02	31.1	33.4	3
106	2	311	3.656	2.692	0.948	7.296	2.75	25	0	22	3	2	2	3.02	2	0.54	10.7	11	0.01	31.3	32.8	3
107	2	906	28.702	10.836	2.356	41.894	4.5	19	0	13	2	2	1	3.18	2	0.375	8.8	3	0.15	27.9	27.8	2
108	2	115	1.562	0.592	0.374	2.528	2.5	28	0	22	0	99	1	4.14	1	0.29	25.6	6.5	0.16	26.6	26.2	3
109	2	635	5.378	1.554	0.404	7.336	3.5	22	1	10	2	1	2	2.73	2	0.17	3.03	10.5	0.09	28.95	30	3
110	2	58	0.182	0.244	0.388	0.814	1	27	0	21	1	1	2	3	2	0.21	0	7.5	0.04	31.9	31	3
111	1	284	4.516	1.008	0.682	6.206	3	25	0	11	2	2	1	2.9	2	0.375	1.33	8	0.29	26.2	28.3	2
112	1	275	4.418	1.546	0.518	6.482	2	28	0	15	1	1	2	2.89	2	0.29	4.2	8	0.19	27.3	27	3
113	1	247	4.222	0.136	0.324	4.682	3	28	0	18	3	2	1	3.09	2	0.458	3.11	9	0.72	29	30.8	2
114	1	768	6.334	9.28	2.048	17.662	4	27	0	26	2	2	2	2.81	2	0.583	1.85	3	0.14	28.7	27.8	2
115	2	58	0.14	0.44	0.682	1.262	2.5	22	0	14	2	2	2	3.23	2	0.625	21.2	4	0.02	28	28.1	3
116	2	462	6.48	4.392	2.318	13.19	4	28	0	0	2	2	2	3	2	0.83	2.97	4.5	0.06	30	29.5	3
117	1	69	1.216	0.384	0	1.6	1.5	6	1	1	2	1	2	3	1	0.83	22.2	16.25	0.04	25.5	26	3
118	2	27	0.15	0.168	0	0.318	1.5	26	2	22	2	1	2	3	2	0.625	0	9	0.03	31.3	31	3
119	2	250	7.238	5.054	2.062	14.354	3	25	0	14	2	2	1	3.16	2	0.5	10	5.5	0.04	31.3	31.9	3
120	1	28	0.126	0	0	0.126	0.5	2	0	2	1	1	2	3	1	0.875	0	8.5	0.15	32.6	32.8	3
121	1	548	8.134	5.646	1.88	15.66	3.5	27	0	14	3	2	1	4.04	1	1.08	19.2	6	0.02	32.8	33.2	2
122	1	587	9.808	4.304	0.966	15.078	4	29	0	2	2	2	1	3.64	2	0.583	14.9	6	0.08	32.9	32.8	3
123	2	516	11.4	7.666	0.904	19.97	3	7	0	6	2	2	1	4.43	1	1.25	35.7	7	0.01	28.2	28.1	3
124	2	45	0.142	0	0	0.142	0.5	29	2	0	2	1	2	2.98	2	0.625	11.3	13.6	1.34	30.7	27.8	3
125	1	253	2.704	0.658	0.678	4.04	2	27	1	15	2	2	1	3.26	2	0.792	20.99	3	0.33	27.4	27.4	3
126	1	301	6.124	4.32	1.404	11.848	3.5	28	1	17	3	2	1	3.11	2	0.83	7.14	16	0.2	30.6	31.7	3
127	2	597	9.456	2.8	0.516	12.772	3	14	0	11	1	2	1	3.64	1	0.375	21.4	14	0.18	29.7	30	3
128	1	148	1.006	0.208	0	1.214	2	26	1	6	2	2	2	3.06	2	0.375	4.5	12.5	0.25	30.6	31.6	2
129	2	426	7.814	2.792	0.834	11.44	3	28	0	13	3	2	2	2.71	2	0.33	17.2	5	0.1	26.9	27.95	3
130	2	27	0.918	0.724	0.858	2.5	1	28	0	17	1	1	2	2.86	2	0.25	0.595	3.5	0.03	25.5	24.2	3
131	1	267	4.414	2.026	0.656	7.096	4	27	0	1	3	2	1	3.02	2	0.5	4.32	5	0.08	28.8	31	3
132	2	507	6.214	4.298	2.078	12.59	3.5	28	0	18	3	2	2	3	2	0.83	7.74	5.5	0.04	31.3	31.1	3
133	2	310	2.754	3.374	1.258	7.386	3	30	0	2	2	2	2	3.01	2	0.375	15.6	12	0.04	29.65	30.5	3
134	2	78	1.068	1.512	1.562	4.142	2	28	0	20	0	99	2	2.96	1	0.08	3.57	10	0.26	29.15	31.4	3
135	2	68	0.946	0.226	0.266	1.438	2	24	0	16	2	1	2	2.94	2	0.5	2.67	3	0.12	31	31.55	3
136	2	204	2.06	1.064	0.982	4.106	2	29	0	11	2	1	2	3.02	2	0.67	1.72	5	0.03	29	28.6	3
